# Pilot Study on the Forehead Skin Microbiome and Short Chain Fatty Acids Depending on the SC Functional Index in Korean Cohorts

**DOI:** 10.3390/microorganisms9112216

**Published:** 2021-10-25

**Authors:** Haryung Park, Karina Arellano, Yuri Lee, Subin Yeo, Yosep Ji, Joontae Ko, Wilhelm Holzapfel

**Affiliations:** 1 Department of Advanced Convergence, Handong Global University, Pohang 37554, Korea; bakhalove7@gmail.com (H.P.); karina.aray@yahoo.com (K.A.); youri4@naver.com (Y.L.); ysji@hempharma.bio (Y.J.); 2 HEM Pharma Inc., Start-Up Incubator, Handong Global University, Pohang 37554, Korea; sbyeo@hempharma.bio; 3 Boaz Medical Hospital, Handong Global University, Pohang 37554, Korea; boaz@handong.edu

**Keywords:** *Cutibacterium*, diversity, dry, moist, short chain fatty acids, skin microbiome, *Staphylococcus*

## Abstract

Dry skin is one of the indicators of a compromised skin barrier. An intact skin barrier is not only important to reserve the hydration within the epidermal tissue but also to protect our skin from environmental stressors and inhibit pathogen invasion; damage to the skin barrier may lead to inflammatory skin diseases. Some microbial metabolites such as short chain fatty acids may inhibit or destroy harmful bacteria and regulate the host immune system. The impact of the skin microbiome and short chain fatty acids on skin barrier function was studied in two groups of 75 participants each. The cohort was equally divided in dry and moist skin types, based on stratum corneum (SC) functionality index (SCFI), reflecting the ratio of transepidermal water loss (TEWL). A dry group represents a low SCFI and a moist group a high SCFI. Compared with the dry skin group, propionate and *Cutibacterium* levels (previously known as *Propionibacterium acnes*) were significantly higher (*p* < 0.001) in the moist group. Levels of *Cutibacterium* were negatively correlated with those of *Staphylococcus* (*p* < 0.0001) in both dry and moist groups. The moist group also had a significantly higher propionate concentration (*p* < 0.001). This study showed that the microbial community and short chain fatty acid concentration may be considered as significant determinants of the SCFI of the skin.

## 1. Introduction

The skin is the largest organ in our body, with a surface area of around 1.8 m^2^ or 25 m^2^ when the diverse “appendage openings” are included [1]. It functions as a first line of defense system protecting our body from environmental stressors and pathogens. The microbiome is represented by around 113 different genera which are unevenly distributed on the surface of the skin [2]. More than a million bacteria cohabiting each 1 cm^2^ of our skin help to regulate the host immune system and strengthen our barrier function [3,4]. Moreover, unique populations of commensal bacteria reside in different parts of our body according to the ecological status of the skin [5]. The stratum corneum (SC), the most outer layer of the epidermis, carries the most important barrier function that protects not only the skin but also the whole body from various diseases. It is already a known fact that our skin becomes vulnerable to various diseases when its barrier function is compromised. Current findings showed that aside from the disruption of the skin barrier, microbial dysbiosis within the skin may also be a cause for atopic dermatitis, xerosis, acne vulgaris, eczema and various other skin diseases [6]. Skin disorders such as atopic dermatitis, psoriasis and different types of eczema are inflammatory diseases accompanied by a disrupted epidermal barrier, low hydration, and high trans-epidermal water loss (TEWL), temperature and pH [7]. Unlike the hydrated skin, dry skin is the underlying cause of many skin problems. It is known to disrupt the integrity of the SC through the impairment of SC cell maturation and reduction in natural moisturizing factors and SC lipids, thereby increasing TEWL [8]. Furthermore, research has proven that the increase in numbers of *Staphylococcus aureus* and decreased *Staphylococcus epidermidis* populations on the skin is associated with atopic dermatitis or dry skin [9]. In a mouse model of atopic dermatitis dysbiosis appeared similar to that reported for atopic dermatitis. *Corynebacterium mastitidis*, *Staphylococcus aureus* and *Corynebacterium bovis* sequentially emerged during the onset of dermatitis. Antibiotics specific for these bacteria minimized dysbiosis and prevented inflammation [10]. 

Recent developments have enabled a deeper scientific understanding of the skin microbiome, and host cutaneous health has corroborated another insight for diagnosis of skin conditions and novel treatment approaches such as skin microbiota modulation. Considering the recognition of the microbiome and its important role in skin health, modulation of the skin microbiome and microbial metabolites using probiotics constitutes a challenging and attractive research area [11]. For example, the skin microbiome and its influence on immune-regulatory functions regarding various skin disorders including atopic dermatitis, has been well explained by previous studies [3]. Yet, specific active molecules such as secondary microbial metabolites need further investigation. Di Marzio et al. [12] reported on the essential role of ceramide in structuring and maintaining the water permeability barrier function of the skin while monitoring the increase in ceramide after short-term topical application of metabolic enzymes such as sphingomyelinase derived from *Streptococcus thermophilus*. Understanding the key microbiota and microbial metabolites influencing the skin health can serve as a scientific basis for dermabiotics (skin probiotics) research. Furthermore, many skin diseases are derived from a broken barrier function which is highly correlated with dry skin [13,14]. A healthy skin barrier is often indicated through the higher ratio of transepidermal water loss (TEWL) which is known as the SC functionality index (SCFI). For this study we therefore divided the participants in two groups representing dry (low SCFI) and moist (high SCFI) skin types. We postulated that differences in the barrier function of the skin may be the result of a modulation of both the skin microbial community and short chain fatty acid (SCFA) concentration.

## 2. Materials and Methods

### 2.1. Study Participants and Sample Collection

This pilot study protocol was approved by the Korean Institutional Bioethics Committee (P01-201808-11-004) and all the procedures were carried out in the Boaz Medical Hospital at Handong Global University. A total of 150 participants were ranged according to their ages from 20 to 50 and were recruited by the Boaz Medical Hospital. Following detailed information, a written consent was signed by all the participants before proceeding with the experimental protocol. People who were suffering of visible skin problems such as acne, inflammation, and redness on the face were excluded. Apart from sampling of the skin microbiome, the participants were tested for their TEWL, hydration and pH. To exclude external contaminations and transient bacteria, participants were asked to carefully wipe off their forehead with a sterile cotton pad drenched in the same type of sterile mild micellar cleansing water (containing 0.05 M NaCl and 0.1% Tween 20) and asked to wait for 20 min. in a humidity and temperature-controlled room (40–50% humidity, 21–22 °C) before the detection of skin parameters (TEWL, hydration, pH) and swab sampling of the skin microbiome and metabolites.

### 2.2. TEWL, Hydration, pH Detection and Skin Microbiome Sampling

The forehead of each volunteer was grouped as either dry or moist skin type using the GPSkin Barrier scanner (GPOWER Inc., Seoul, South Korea) which measures the hydration and TEWL level of the skin. Dermalab^®^ (CortexTechnology, Handund, Denmark) transepidermal water loss (TEWL) and hydration probes were used to double check the skin trans-epidermal water loss and hydration levels of the skin. Hanna Instruments Skin pH meter (Woonsocket, RI, USA) was used to check the skin pH as well. For the sampling of the skin microbiome an ESwab™ (Copan Diagnostics, Brescia, Italy) was premoistened in sterile 0.05 M NaCl and 0.1% Tween 20 and used to sample the forehead of each volunteer for 30 s with the same pressure. The swab was released into the provided liquid amie (solution in a vial to collect and preserve the swab until analysis) and stored at −80 °C until further analysis.

### 2.3. Short Chain Fatty Acid Analysis

The short chain fatty acids (SCFA) were extracted using the extraction buffer containing ammonium sulfate and sodium dihydrogen phosphate and 8% meta-phosphoric acid in a ratio of (3.7:1), adjusted to pH 2.0, as suggested by Fiorini et al. [15]. Each skin sample contained in liquid amie was mixed with the buffer in a 1:1 ratio and the SCFA were detected using gas chromatography (GC, Agilent 7890, Santa Clara, CA, USA) inserted with the headspace (oven temp. 75 °C, eq. time 10 min.). The INNOWAX 30 m × 0.23 mm column (Agilent, Santa Clara, CA, USA) was used running from 100 °C to 180 °C in 25 °C/m at 27.1 psi with a constant flow rate of the helium carrier gas at 1 mL/min. detected through a flame ionized detector (FID). A volatile fatty acid mixture (Supelco, 46975-U, Saint Louis, MI, USA) was used to create a standard curve for the short chain fatty acids, and the samples were detected using the FID. 

### 2.4. Bacterial DNA Extraction

The bacterial DNA was extracted using the QIAmp DNA mini kit (Qiagen, Valencia, CA, USA) with a few modifications. A total of 200 μL of the liquid amie, containing the skin swab sample, was mixed with 0.3 g of 0.01 mm sterile zirconia beads with 500 μL of stool lysis buffer (Qiagen, Valencia, CA, USA)) in a 2 mL bead-beating tube and homogenized for 3 min. using the TissueLyser II (Thermo Scientific, Waltham, MA, USA) to disintegrate the bacterial cell walls. DNA extraction procedures were performed according to the QIAmp DNA Mini Kit manufacturer’s instructions with slight modifications.

### 2.5. Library Preparation and Microbiome Analysis

The bacterial DNA was diluted with 10 mM of Tris–HCl pH 8.5 buffer to 5 ng/μL prepared according to the Illumina 16S metagenomics sequencing library protocol. The 16S rRNA V3- V4 region was amplified using the following amplicon primers: 16S Amplicon PCR Forward Primer 5′TCG TCG GCA GCG TCA GAT GTG TAT AAG AGA CAG CCT ACG GGN GGC WGC AG′3 and 16S Amplicon PCR Reverse Primer 5′GTC TCG TGG GCT CGG AGA TGT GTA TAA GAG ACA GGA CTA CHV GGG TAT CTA ATC C′3 [16]. The amplified samples with linker primers were then barcoded using the dual indexing method involving the Nextera XT kit (Illumina, San Diego, CA, USA). The final products were normalized and pooled using PicoGreen, and the size of the libraries were verified using the LabChip GX HT DNA High Sensitivity Kit (PerkinElmer, Waltham, MA, USA) and performed on an Illumina Miseq platform. The barcode, linker, and primer sequences were then removed from the original sequencing reads and replaced with sample names.

The removed reads were merged by their paired ends using FLASH v 1.2.11 [17]. The merged reads containing two or more ambiguous nucleotides, those with a low-quality score (average score < 20), and reads shorter than 300 base pairs, were filtered out. Potential chimeric sequences were detected using the Bellerophon method [18]. The pre-processed reads from each sample were used to calculate the number of operational taxonomic units (OTUs). The number of OTUs was determined by clustering the sequences from each sample using a 97% sequence identity cut-off to Green Genes database and the taxonomic profiling the alpha diversity (Shannon, Simpson, Chao1, Observed_OTU and PD_whole_tree), and beta diversity (weighted and unweighted Unifrac PCoA plots) of each group were analyzed and visualized using the macQIIME software (v.1.9.1) [19]. The skin microbiome was analyzed using the same tool except that the taxonomic profiling was retrained using the RDP Classifier. The R software (version 3.14) and pyloseq and ggplot2 packages were used to visualize the NMDS (non-metric multidimensional scaling) dissimilarity plot based on the Bray-Curtis and LefSe analysis depending on the LDA Score filter value of 5. 

### 2.6. Statistical Analysis

The skin microbiome data were compared using an unpaired parametric *t*-test with Welch correction or a one-way ANOVA was used to compare the differences between different skin types and sexes using the GraphPad Prism 9.0 (GraphPad Software, San Diego, CA, USA). All the graphs are presented as the mean value and standard deviation (SD). The statistical analysis of the LDA score and PERMANOVA was performed using the R software (version 3.14).

## 3. Results

### 3.1. TEWL and Hydration of Moist and Dry Skin

One hundred and fifty volunteers were checked for their skin hydration, trans-epidermal water loss (TEWL), and pH and grouped into moist and dry skin depending on their hydration to TEWL ratio provided by the GPSkin Barrier detector [15]. The measurements of the TEWL and hydration were reconfirmed using the Dermalab probes. There were 75 people in each of the dry and moist groups (Figure S1C). Most of the participants were in their 20–30s (Figure S1B) and 56% of them were female (Figure S1A). 

**Figure 1:**
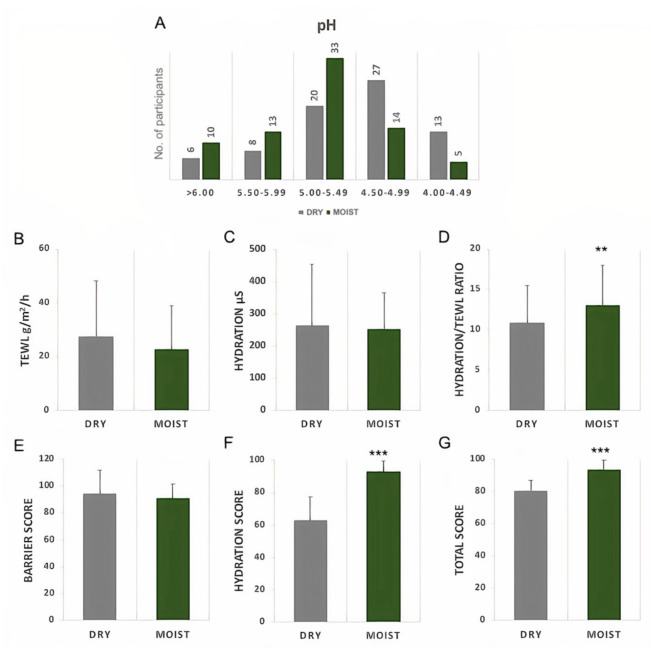
Comparison of skin barrier based on TEWL, hydration and pH. (**A**) pH; (**B**) TEWL (trans-epidermal water loss); (**C**) hydration level; (**D**) hydration/TEWL ratio; (**E**) barrier score; (**F**) hydration score; (**G**) total score. (**A**–**C**) measurements provided by the Dermalab combo probes. (**E**–**G**) Provided score from the GPSkin Barrier detector. Data are presented with mean and standard deviation and analyzed with unpaired parametric *t*-test with Welch correction compared with the dry group. ** *p* < 0.01, *** *p* < 0.001.

Both the moist and dry groups had the highest number of people in the healthy pH range of 4.5–5.5 (Figure 1A). The TEWL and hydration levels were measured with two different devices, the Dermalab combo probe (Figure 1B–D) and the GPSkin Barrier detector (Figure 1E–G) [20]. The moist group showed a slight decrease in the TEWL; however, it was not significant in any device (Figure 1B,E). Although no differences in the hydration level were shown through detection by the Dermalab probe, a significantly higher hydration score was measured by the GPSkin detector in the moist group (Figure 1C,F). The moist group did not show significant differences in the decrease and increase in TEWL and hydration levels, respectively, with the Dermalab probe, yet the ratio of hydration/TEWL increased significantly. This suggests that the moist group had a lower TEWL even with possibly similar hydration values as the dry group, indicating a more intact skin barrier (Figure 1D). The results were confirmed by the total score in the GPSkin Barrier detector (Figure 1G). Furthermore, compared with the male participants, there were more female participants with moist skin (Figure S1D,E).

### 3.2. Differences in the Skin Microbiota Related to the Skin-Type and Sex

**Figure 2:**
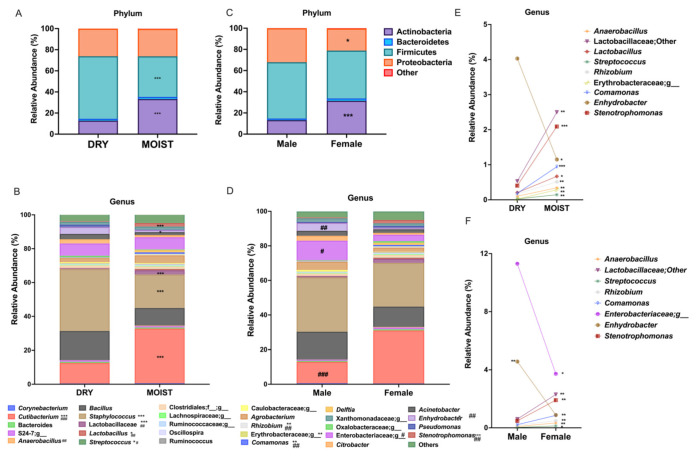
Comparison of the microbiota associated with skin-type and gender. (**A**,**C**) Phylum level. (**B**,**D**–**F**) genus level. Data were analyzed with unpaired parametric *t*-test comparing moist and dry group * *p* < 0.05, ** *p* < 0.01, *** *p* < 0.001 and comparing male and female ^#^ *p* < 0.05, ^##^ *p* < 0.01, ^###^ *p* < 0.001.

There was a clear difference in the microbiota of the moist and dry skins at both the phylum and genus level. Among the four main skin phyla there was a significantly higher relative abundance of Actinobacteria and lower numbers of Firmicutes in the moist group compared with the dry group (Figure 2A). Moreover, relative to the dry group, the moist group was associated with a significantly lower abundance of the genus *Staphylococcus* (phylum Firmicutes) and comparatively high *Cutibacterium* levels (phylum Actinobacteria) (Figure 2B). The moist group also had significantly higher *Lactobacillus*, *Lactobacillaceae*, *Anaerobacillus*, *Streptococcus*, *Rhizobium*, *Erythrobacteraceae*, *Comamonas* and *Stenotrophomonas* populations and significantly lower numbers of *Enhydrobacter* (Figure 2E). The effect size of the microbial communities was shown through the LDA analysis indicating that *Cutibacterium acnes*, Lactobacillales, and *Agrobacterium* were significantly represented in the moist group compared with the significantly higher levels of Bacillales and *Staphylococcus* spp. in the dry group (Figure S3A). 

When comparing sexes, the female group showed closer similarity to the moist group with a significantly higher relative abundance of Actinobacteria while that of the Proteobacteria was significantly lower (Figure 2C). At the genus level, the female group was characterized by a significantly higher abundance of *Cutibacterium* (Figure 2D), *Anaerobacillus*, *Lactobacillaceae*, *Streptococcus*, *Rhizobium*, *Comamonas* and *Stenotrophomonas* and a significantly lower representation of the family *Enterobacteriaceae* and the genus *Enhydrobacter* compared with the male group (Figure 2F). 

### 3.3. Differences in the Skin Microbiota Diversity According to Skin-Type and Sex

**Figure 3:**
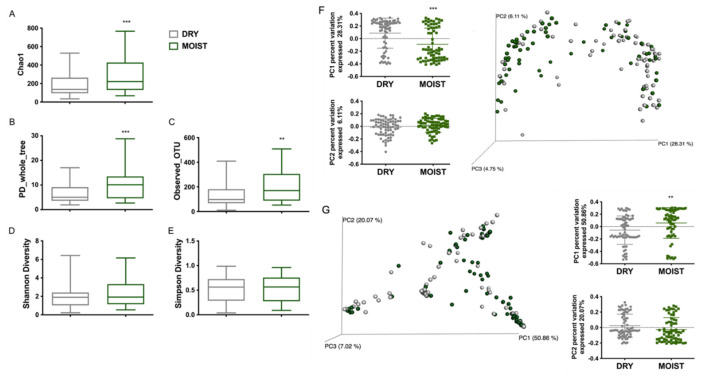
Diversity of the microbiota of dry and moist skin. (**A**) Chao 1. (**B**) PD_whole_tree, comparing alpha diversity. (**C**) observed OTUs. (**D**) Shannon Diversity. (**E**) Simpson Diversity. (**F**) Unweighted PCoA plot and PC1 and PC2 percent variation. (**G**) Weighted PCoA plot and PC1 and PC2 percent variation. (**A**–**E**) Alpha diversity. (**F**–**G**) Beta-diversity. Data were analyzed with unpaired parametric *t*-test with Welch correction compared with dry group. ** *p* < 0.01, *** *p* < 0.001.

The moist group had a significantly higher richness (chao1, PD_whole_tree, observed_OTUs) but no difference in the evenness (Shannon and Simpson diversity) of the alpha diversity compared with the dry group (Figure 3A–E). Similar to the moist group the female group had a significantly higher alpha diversity richness (Figure S2A–C) compared with the male group and also a significantly higher evenness when compared using the Shannon diversity index (Figure S2D).

The beta-diversity of the microbiota associated with dry and moist skin was clearly different through both the weighted and unweighted PCoA plots (Figure 3F,G). There was a significant difference in the clustering of both the moist and dry groups through the PC1 percent variation (28.31%, 50.86%) axis of unweighted and weighted PCA plots, respectively (Figure 3F). Moreover, there was a noticeable difference between the moist and dry skin groups in the multivariate PERMANOVA analysis of the Bray-Curtis dissimilarity ordination (Figure S3B). The change in the beta-diversity was also calculated using the sex category. Comparing females to males, a significant difference in the weighted beta-diversity was illustrated by both the PC1 percent variation (50.86%) and PC2 percent variation (20.07%) (Figure S2F). Moreover, the unweighted beta-diversity portrayed a distinct difference in the clustering of both groups through the PC1 percent variation (28.31%) (Figure S2G). 

### 3.4. Differences in the Skin Microbiota Diversity According to Skin-Type within Each Sex Group

**Figure 4:**
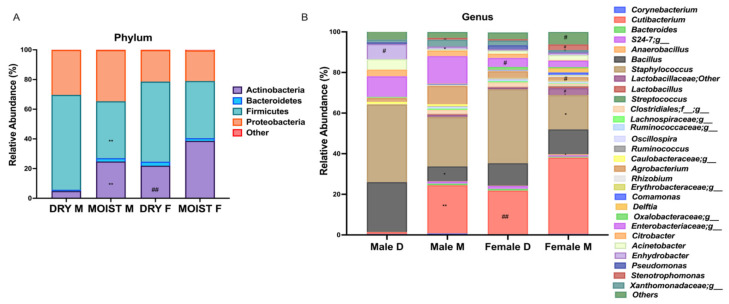
Comparison of the microbiota associated with moist and dry forehead skins with regard to gender differences. (**A**) Phylum level. (**B**) Genus level. Data were analyzed with unpaired parametric *t*-test compared between skin types within each sex * *p* < 0.05, ** *p* < 0.01 and compared between sex for each skin type ^#^ *p* < 0.05, ^##^ *p* < 0.01. M = moist, D = dry.

The difference between the sdex-specific moist and dry groups were also analyzed since the female group had a higher moist population (56%) compared with the male group (42%) (Figure S1D,E). Within the phylum, the abundance of the Actinobacteria was significantly lower within the dry male group compared with the moist male group and dry female group (Figure 4A). The dry male group also had a significantly higher Firmicutes population compared to the moist male group (Figure 4A). At the more specified genus and family levels, the male moist group showed a significantly higher abundance of *Cutibacterium* and *Stenotrophomonas* and significantly lower *Bacillus*, *Lactobacillaceae*, *Ruminococcaceae*, *Agrobacterium*, *Comamonas*, *Strenophomonas* and *Enhydrobacter* compared with the male dry group (Figure 4B). When compared with the female dry group, the dry male group had significantly higher levels of *Enhydrobacter*, *Strenophomonas*, *Comamonas*, *Erythrobaceraceae*, *Ruminococcaceae* and *Enterobacteriaceae* and significantly lower levels of *Cutibacterium* spp. (Figure 4B). In between the dry and moist group of the female population, there was surprisingly no significant difference in *Cutibacterium* levels; however, the levels of *Staphylococcus* were significantly lower while te hlevels of *Anaerobacillus*, *Streptococcus*, *Rhizobium*, *Erythrobacteraceae*, *Comamonas*, *Lactobacillaceae* and *Stenotrophomonas* were higher in the moist group compared with the dry group (Figure 4B). 

The alpha diversity of both the dry and moist communities differed significantly within each sex group, with moist groups have an overall higher richness represented by Chao1, PD_whole_tree and Observed OTU (Figure S4A–C). Moreover, compared with the male dry group the female dry group showed significantly higher richness in the alpha diversity (Figure S4B,C). For the unweighted beta-diversity of the moist (Figure S4F) and dry (Figure S4E) communities a significant sex difference as detected within each skin type. 

### 3.5. Differences in the Skin Short Chain Fatty Acid (SCFA) Profile

**Figure 5:**
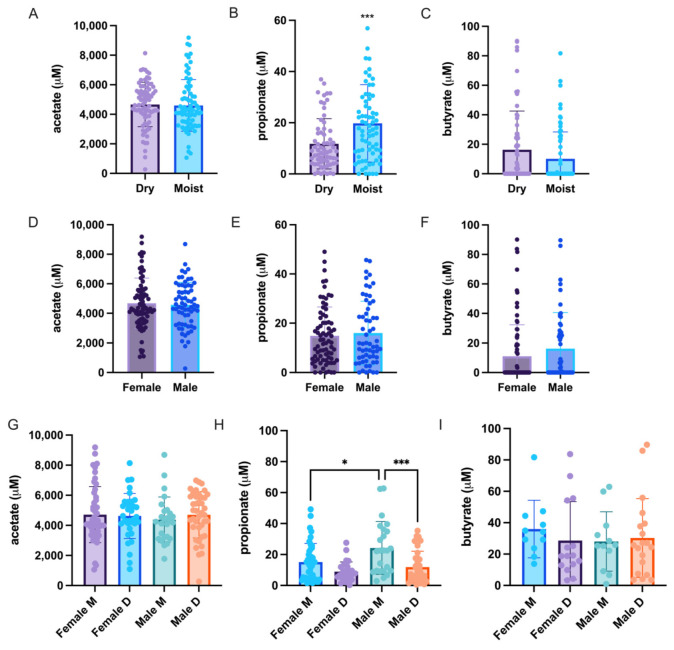
Comparison of sex influence on the short chain fatty acids (SCFAs) of dry and moist skins. (**A**,**D**,**G**) Acetate. (**B**,**E**,**H**) Propionate. (**C**,**F**,**I**) Butyrate. Data were analyzed with unpaired parametric *t*-test with Welch correction (dry vs. moist and female vs. male) and one-way ANOVA was used to analyze the significance between the skin types within each sex. * *p* < 0.05, *** *p* < 0.001.

SCFAs are important metabolic byproducts of bacterial fermentation in the large intestine of the human host. It was therefore attempted to check for a possible correlation between the amount of certain SCFA producing bacteria such as *Cutibacterium* and *Staphylococcus* within the skin. A significantly higher propionate production can be detected within the moist group when compared with the dry group (Figure 5B). In most of the samples very low to zero levels of butyrate were found, while only insignificant acetate or butyrate levels were detected in all groups (Figure 5). The propionate levels significantly differed between the male moist and dry group and female moist and male moist group (Figure 5H). Since *Cutibacterium* produces propionate [21] and some *Staphylococcus* species have been reported to produce butyrate [22], we performed Pearson’s correlation to check for a positive correlation between the respective samples; however, there was no significant correlation between the two parameters for bacteria nor SCFA (Figure S5A). 

## 4. Discussion

The barrier integrity of the skin is the most important initial parameter for protection of the skin against adverse environmental influences and the development of various diseases. In addition, it acts as the “first line of defense” for maintaining the stability and integrity of the human host [23,24]. The moisture level of the skin is usually detected by the hydration level of the skin surface whereas its barrier integrity can be measured by TEWL as an indication of the level of hydration within the skin [25,26,27]. The participants within this study were grouped into moist and dry according to their transepidermal water loss and hydration level initially detected with the GPSkin Barrier detector and confirmed with the Dermalab combo probe. The moist group had a significantly higher hydration score and a lower TEWL level represented by the SC functional index (hydration/TEWL ratio) (Figure 1). These data indicate that although the hydration levels were not significantly different the water loss on the surface of the skin was lower, suggesting a stronger barrier integrity [28]. Various studies have shown that both the moisture level and barrier integrity play a decisive role in preventing allergic diseases such as atopic dermatitis, psoriasis, Netherton syndrome and xerosis, all of which are associated with dry, scaly skin and a breached barrier [29,30,31]. Moreover, these allergic diseases, associated with dry skin and weak barrier function, have been correlated with dysbiosis in the skin microbiome, especially an overload of *Staphylococcus* [11,32,33,34,35]. Against this rationale, the microbial communities of the dry (low SC functionality index) and moist (high SC functionality index) skins of participants with healthy skins were detected during this study to determine whether there were changes in the microbiome in a dry and weakened skin barrier even without any subsequent skin problems.

Our data suggests a distinct microbiome for dry and moist skins; clustering by beta-diversity showed microbial populations to be significantly different (Figure 3F,G). The population of the genus *Staphylococcus* was significantly higher on the dry skin, whereas significantly higher *Cutibacterium* and significantly lower *Staphylococcus* populations were detected on the moist skin (Figure 2A,B). The breach of the barrier causes a greater water loss in the skin creating a dry environment favored by *Staphylococcus*. Furthermore, the moist group had a higher bacterial richness compared with the dry group (Figure 3A–C). The positive correlation of moisture level with *Cutibacterium* and lower abundance of *Staphylococcus* was shown in a study on the skin microbiome of rural and urban Chinese populations [36]. Although diversity is still open to interpretation, depending on the shift of the microbiome, a few studies have shown that its decrease within skin diseases and can be correlated with an overload of a certain population such as *Staphylococcus aureus* in atopic dermatitis compared with a healthy population [37,38]. Moreover, compared with healthy skin, a lower diversity was also reported for dry and itchy skin [39]. Our study showed similar results for the moist and dry groups within the different sexes (Figure 4 and Figures S2–S4). However, since there were more moist participants within the female group (Figure S1) we divided the skin types within the sex to eliminate differences on this basis. The male dry group had a significantly higher *Bacillus* and significantly lower *Cutibacterium* population compared with all the moist groups and even the dry female group (Figure 4B). This result differs from data generated by the skin study of the Chinese population in which male participants had a significantly higher abundance of *Cutibacterium* compared with the females when tested in the glabella [36]. A recent study on the skin microbiome profile on 51 healthy Korean volunteers and in dependance of the sex and age, reported a significantly high abundance of *Staphylococcus* and *Corynebacterium* in male participants and *Lactobacillus* in female participants [40]. This was also true within our study except for the abundance of *Corynebacterium*; rather there were significant levels of abundance of *Enhydrobacter* and *Enterobacteriaceae* in the male group compared with the female group (Figure 2F). 

A few publications have reported on the sebum and hydration levels of the cheek and forehead [41,42] or the sex specific difference of the skin [40,43,44] and their relationship to the microbiome. However, as far as it is known, no publications have yet investigated the correlation of the skin microbiome and its overall SCFA profile. The SCFAs, especially acetate, propionate, and butyrate, are the main metabolites produced by bacterial fermentation in the human microbiome [45]. The SCFA have been known to play a crucial role in ameliorating metabolic and inflammatory diseases in the gut microbiome [46,47]. A few in vitro studies investigated the effects of SCFAs produced by *Cutibacterium acnes* (previously known as *Propionibacterium acnes*) and *Staphylococcus epidermidis* on the skin [22,48,49,50] or by using the SCFA directly [51,52]. In our study, the levels of propionate were significantly higher in the moist group compared with the dry group (Figure 5B) which also had significantly higher *Cutibacterium* levels (Figure 2B). These levels were also significantly higher in the male moist group compared with both the male dry and female moist groups (Figure 5H). This was not proportionate to the *Cutibacterium* levels in each group since the female moist group had the highest *Cutibacterium* abundance compared with the male moist group, yet the male moist group had a significantly higher propionate level (Figure 5B). However, there was no significant correlation between the levels of propionate and *Cutibacterium* abundance (Figure S5A). Nor was there any correlation between the butyrate level and *Staphylococcus* abundance (Figure S5B). SCFAs have been shown to have anti-inflammatory effects in monocytes whereas in keratinocytes and sebocytes showed the opposite effect of increasing the inflammatory cytokine expression due to the inhibition of histone acetylation and histone deacetylase (HDAC) which is responsible for the decrease in inflammatory cytokines; this was also confirmed for propionate produced by *C. acnes* [50,51]. It has been shown that propionate produced by *C. acnes* inhibit the biofilm formation of some *Staphylococcus* species such as *S. epidermidis* and *S. aureus* to a lesser degree [48]. Within our study, there was a significant negative correlation between *Cutibacterium* and *Staphylococcus* (Figure S5C) and a slight, but not significant, negative correlation between propionate and *Staphylococcus* (Figure S5D). 

Most allergic skin diseases, including acne, have a strong correlation with the overload of *Staphylococcus*, especially *Staphylococcus aureus* [53,54,55]. Dry skin, which is the hallmark of a weakened barrier, usually leads to a higher skin pH and these factors combined may favor the presence and growth of *Staphylococcus* species [2]; this was also shown by our data in the dry groups of both sexes (Figure 4B). Moreover, Chng et al. [56] found the presence of, e.g., *Staphylococcus*, in certain microbiomes to have a cross-modulating effect with the host immune system which may trigger the severity of a certain disease. Moisturizing the skin with emollients is known to reduce and prevent inflammatory responses in allergic reactions and dry skin barrier disorders [57,58]. Hydration and barrier integrity may play a role in creating a favorable environment to inhibit pathogen invasion through the stabilization of the commensal microbiome. Furthermore, the postbiotic materials of bacteria such as SCFAs may act as a good immune regulator in aiding host defense against pathogens and or ameliorating inflammation and hyperpigmentation due to UV damage [22,48,49]. Our study has showed a significant difference in diversity and domination in the microbial communities of dry and moist forehead skins. 

## 5. Conclusions

In our study we compared differences in the microbiome and short chain fatty acids of moist and dry groups. Compared with the dry group significantly higher levels of *Cutibacterium,* propionic acid, and alpha diversity and a lower abundance of *Staphylococcus* were detected in the moist group. The PERMANOVA test showed a significant dissimilarity between the two groups. The moist group was characterized by a higher abundance of *Cutibacterium*, *Agrobacterium*, *Rhizobiaceae* and Lactobacillales, whereas relatively high levels of *Staphylococcus* were found typical of the dry group. Significant differences in the forehead skin microbiome and propionic acid levels appeared to be related to the SC functionality index. 

## Data Availability

The data presented in this study are available on request from the corresponding author or Haryung Park (hrpark@hempharma.bio).

## Supplementary Materials

The following are available online: https://www.mdpi.com/article/10.3390/microorganisms9112216/s1, Figure S1: Information on the 150 volunteers for the skin studies. (A) Sex. (B) Age. (C) Skin barrier type. (D) Female skin barrier ratio. (E) Male skin barrier ratio. Figure S2: Comparison of sex influence on the diversity of the forehead skin microbiota. (A) Chao 1. (B) PD_whole_tree. (C) Observed_OTUs. (D) Shannon Diversity. (E) Simpson Diversity. (F) Weighted PCoA plot and PC1 and PC2 percent variation. (G) Unweighted PCoA plot and PC1 and PC2 percent variation. (A–E) Alpha diversity. (F–G) Beta-diversity. Data were analyzed with unpaired parametric *t*-test with Welch correction compared between sexes. * *p* < 0.05, ** *p* < 0.01, *** *p* < 0.001. Figure S3: Effect of size and differences in microbial communities on dry and moist skins. (A) Linear discriminant analysis (LDA) and effect Size (LefSe) analysis of microbial communities on dry and moist forehead skins. Positive LDA scores (red bars) are enriched in the dry group and negative LDA scores (blue bars) are enriched in the moist group. The microbial taxa and functions shown in the Figure have a LDA score higher than 5. (B) Bray-Curtis based NMDS results for microbial community groups of dry and moist skins (PERMANOVA, df:1, SS: 0.06199, MS:0.061990, F:5.3909, *p*-value < 0.019). Figure S4: Comparison of sex influence on the diversity of the forehead skin microbiota of dry and moist skin. (A) Chao 1. (B) PD_whole_tree. (C) Observed_OTUs. (D) Shannon Diversity. (E) Simpson Diversity. (F) Dry group unweighted PCoA plot and PC1 and PC2 percent variation. (G) Moist group unweighted PCoA plot and PC1 and PC2 percent variation. (A–E) alpha diversity. (F–G) beta-diversity. Data were analyzed with unpaired parametric *t*-test with Welch correction compared with dry group * *p* < 0.05, ** *p* < 0.01, *** *p* < 0.001 and between sex # *p* < 0.05. Figure S5: Correlation between the two major bacterial genera and short chain fatty acids (SCFA) of the forehead skin of a cohort of 150 Korean participants. (A) Propionate vs. *Cutibacterium*. (B) Butyrate vs. *Staphylococcus*. (C) *Staphylococcus* vs. *Cutibacterium*. (D) Propionate vs. *Staphylococcus*. Data were analyzed by calculating the Pearson’s correlation coefficient.

## References

[1] Gallo R.L. (2017). Human skin is the largest epithelial surface for interaction with microbes. J. Investig. Dermatol..

[2] Rojo D., Méndez-García C., Raczkowska B.A., Bargiela R., Moya A., Ferrer M., Barbas C. (2017). Exploring the human microbiome from multiple perspectives: Factors altering its composition and function. FEMS Microbiol. Rev..

[3] Belkaid Y., Segre J.A. (2014). Dialogue between skin microbiota and immunity. Science.

[4] Sanford J.A., Gallo R.L. (2013). Functions of the skin microbiota in health and disease. Sem. Immunol..

[5] Grice E.A., Segre J.A. (2011). The skin microbiome. Nat. Rev. Microbiol..

[6] Prescott S.L., Larcombe D.L., Logan A.C., West C., Burks W., Caraballo L., Levin M., Van-Etten E., Horwitz P., Kozyrskyj A. (2017). The skin microbiome: Impact of modern environments on skin ecology, barrier integrity, and systemic immune programming. World Allergy Org. J..

[7] Montero-Vilchez T., Segura-Fernández-Nogueras M.V., Pérez-Rodríguez I., Soler-Gongora M., Martinez-Lopez A., Fernández-González A., Molina-Leyva A., Arias-Santiago S. (2021). Skin barrier function in psoriasis and atopic dermatitis: Transepidermal water loss and temperature as useful tools to assess disease severity. J. Clin. Med..

[8] Rawlings A.V., Matts P.J., Anderson C.D., Roberts M.S. (2008). Skin biology, xerosis, barrier repair and measurement. Drug Discov. Today Dis. Mech..

[9] Byrd A.L., Deming C., Cassidy S.K., Harrison O.J., Ng W.I., Conlan S., Belkaid Y., Segre J.A., Kong H.H., NISC Comparative Sequencing Program (2017). Staphylococcus aureus and Staphylococcus epidermidis strain diversity underlying pediatric atopic dermatitis. Sci. Transl. Med..

[10] Kobayashi T., Glatz M., Horiuchi K., Kawasaki H., Akiyama H., Kaplan D.H., Kong H.H., Amagai M., Nagao K. (2015). Dysbiosis and *Staphylococcus aureus* colonization drives inflammation in atopic dermatitis. Immunity.

[11] Grice E.A. (2014). The skin microbiome: Potential for novel diagnostic and therapeutic approaches to cutaneous disease. Semin. Cutan. Med. Surg..

[12] Di Marzio L., Cinque B., Cupelli F., De Simone C., Cifone M.G., Giuliani M. (2008). Increase of skin-ceramide levels in aged subjects following a short-term topical application of bacterial sphingomyelinase from *Streptococcus thermophilus*. Int. J. Immunopathol. Pharmacol..

[13] Bouwstra J.A., Ponec M. (2006). The skin barrier in healthy and diseased state. Biochim. Biophys. Acta.

[14] Yosipovitch G. (2004). Dry skin and impairment of barrier function associated with itch–new insights. Int. J. Cosmet. Sci..

[15] Fiorini D., Boarelli M.C., Gabbianelli R., Ballini R., Pacetti D. (2016). A quantitative headspace–solid-phase microextraction–gas chromatography–flame ionization detector method to analyze short chain free fatty acids in rat feces. Anal. Biochem..

[16] Amplicon P.C.R., Clean-Up P.C.R., Index P.C.R. (2013). 16S Metagenomic Sequencing Library Preparation.

[17] Magoč T., Salzberg S.L. (2011). FLASH: Fast length adjustment of short reads to improve genome assemblies. Bioinformatics.

[18] Hayes M., Li J. (2013). Bellerophon: A hybrid method for detecting interchromo-somal rearrangements at base pair resolution using next-generation sequencing data. BMC Bioinform..

[19] Caporaso J.G., Kuczynski J., Stombaugh J., Bittinger K., Bushman F.D., Costello E.K., Fierer N., Pena A.G., Goodrich J.K., Gordon J.I. (2010). QIIME allows analysis of high-throughput community sequencing data. Nat. Methods.

[20] Ye L., Wang Z., Li Z., Lv C., Man M.Q. (2019). Validation of GPSkin Barrier^®^ for assessing epidermal permeability barrier function and stratum corneum hydration in humans. Skin Res. Technol..

[21] Tax G., Urbán E., Palotás Z., Puskás R., Kónya Z., Bíró T., Kemény L., Szabo K. (2016). Propionic acid produced by *Propionibacterium acnes* strains contributes to their pathogenicity. Acta Derm. Venereol..

[22] Keshari S., Balasubramaniam A., Myagmardoloonjin B., Herr D.R., Negari I.P., Huang C.M. (2019). Butyric acid from probiotic *Staphylococcus epidermidis* in the skin microbiome down-regulates the ultraviolet-induced pro-inflammatory IL-6 cytokine via short-chain fatty acid receptor. Int. J. Mol. Sci..

[23] Smith W. (1999). Stratum corneum barrier integrity controls skin homeostasis. Int. J. Cosmet. Sci..

[24] Elias P.M., Wakefield J.S. (2014). Mechanisms of abnormal lamellar body secretion and the dysfunctional skin barrier in patients with atopic dermatitis. J. Allergy Clin. Immunol..

[25] Van Rensburg S.J., Franken A., Du Plessis J.L. (2019). Measurement of transepidermal water loss, stratum corneum hydration and skin surface pH in occupational settings: A review. Skin Res. Tech..

[26] Shah D.K., Khandavilli S., Panchagnula R. (2008). Alteration of skin hydration and its barrier function by vehicle and permeation enhancers: A study using TGA, FTIR, TEWL and drug permeation as markers. Methods Find. Exp. Clin. Pharmacol..

[27] Thune P., Nilsen T., Hanstad I.K., Gustavsen T., Lövig H.D. (1988). The water barrier function of the skin in relation to the water content of stratum corneum, pH and skin lipids. The effect of alkaline soap and syndet on dry skin in elderly, non-atopic patients. Acta Derm. Venereol..

[28] Du Plessis J.D., Stefaniak A., Eloff F., John S., Agner T., Chou T.C., Nixon R., Steiner M., Franken A., Kudla I. (2013). International guidelines for the in vivo assessment of skin properties in non-clinical settings: Part 2. transepidermal water loss and skin hydration. Skin Res. Technol..

[29] Hudson T.J. (2006). Skin barrier function and allergic risk. Nat. Gen..

[30] Guzik T.J., Bzowska M., Kasprowicz A., Czerniawska-Mysik G., Wójcik K., Szmyd D., Adamek-Guzik T., Pryjma J. (2005). Persistent skin colonization with *Staphylococcus aureus* in atopic dermatitis: Relationship to clinical and immunological parameters. Clin. Exp. Allergy.

[31] Kong H.H., Oh J., Deming C., Conlan S., Grice E.A., Beatson M.A., Nomicos E., Polley E.C., Komarow H.D., NISC Comparative Sequence Program (2012). Temporal shifts in the skin microbiome associated with disease flares and treatment in children with atopic dermatitis. Genome Res..

[32] Tett A., Pasolli E., Farina S., Truong D.T., Asnicar F., Zolfo M., Beghini F., Armanini F., Jousson O., De Sanctis V. (2017). Unexplored diversity and strain-level structure of the skin microbiome associated with psoriasis. NPJ Biofilms Microbiomes.

[33] Williams M.R., Cau L., Wang Y., Kaul D., Sanford J.A., Zaramela L.S., Khalil S., Butcher A.M., Zengler K., Horswill A.R. (2020). Interplay of Staphylococcal and host proteases promotes skin barrier disruption in Netherton syndrome. Cell Rep..

[34] Rawlings A.V., Harding C.R. (2004). Moisturization and skin barrier function. Dermatol. Ther..

[35] Cork M.J. (1997). The importance of skin barrier function. J. Dermatol..

[36] Ying S., Zeng D.N., Chi L., Tan Y., Galzote C., Cardona C., Lax S., Gilbert J., Quan Z.X. (2015). The Influence of age and gender on skin-associated microbial communities in urban and rural human populations. PLoS ONE.

[37] Baurecht H., Rühlemann M.C., Rodríguez E., Thielking F., Harder I., Erkens A.S., Stolzl D., Ellinghaus E., Hotze M., Lieb W. (2018). Epidermal lipid composition, barrier integrity, and eczematous inflammation are associated with skin microbiome configuration. J. Allergy Clin. Immunol..

[38] Wallen-Russell C., Wallen-Russell S. (2017). Meta-analysis of skin microbiome: New link between skin microbiota diversity and skin health with proposal to use this as a future mechanism to determine whether cosmetic products damage the skin. Cosmetics.

[39] Futterer T., Tierney N., Rush A., Meyer K., Capone K. (2019). Assessment of skin microbiome diversity and skin health in dry skin and dry, itchy skin: A bilateral, controlled clinical trial using oat-containing lotions and wash. J. Am. Acad. Dermatol..

[40] Kim J.H., Son S.M., Park H., Kim B.K., Choi I.S., Kim H., Huh C.S. (2021). Taxonomic profiling of skin microbiome and correlation with clinical skin parameters in healthy Koreans. Sci. Rep..

[41] Mukherjee S., Mitra R., Maitra A., Gupta S., Kumaran S., Chakrabortty A., Majumder P.P. (2016). Sebum and hydration levels in specific regions of human face significantly predict the nature and diversity of facial skin microbiome. Sci. Rep..

[42] Lee H.J., Jeong S.E., Lee S., Kim S., Han H., Jeon C.O. (2018). Effects of cosmetics on the skin microbiome of facial cheeks with different hydration levels. Microbiologyopen.

[43] Ross A.A., Doxey A.C., Neufeld J.D. (2017). The skin microbiome of cohabiting couples. MSystems.

[44] Ma Z., Li W. (2019). How and why men and women differ in their microbiomes: Medical ecology and network analyses of the microgenderome. Adv. Sci..

[45] Silva Y.P., Bernardi A., Frozza R.L. (2020). The role of short-chain fatty acids from gut microbiota in gut-brain communication. Front. Endocrinol..

[46] Blaak E.E., Cnafora E.E., Theis S., Frost G., Groen A.K., Mithieux G., Nauta A., Scott K., Stahl B., van Harsselaar J. (2020). Short chain fatty acids in human gut and metabolic health. Benef. Microbes.

[47] Tan J., McKenzie C., Potamitis M., Thorburn A.N., Mackay C.R., Macia L. (2014). The role of short-chain fatty acids in health and disease. Adv. Immunol..

[48] Nakamura K., O’Neill A.M., Williams M.R., Cau L., Nakatsuji T., Horswill A.R., Gallo R.L. (2020). Short chain fatty acids produced by *Cutibacterium acnes* inhibit biofilm formation by *Staphylococcus epidermidis*. Sci. Rep..

[49] Kao H.J., Wang Y.H., Keshari S., Yang J.J., Simbolon S., Chen C.C., Huang C.M. (2021). Propionic acid produced by *Cutibacterium acnes* fermentation ameliorates ultraviolet B-induced melanin synthesis. Sci. Rep..

[50] Sanford J.A., O’Neill A.M., Zouboulis C.C., Gallo R.L. (2019). Short-chain fatty acids from *Cutibacterium acnes* activate both a canonical and epigenetic inflammatory response in human sebocytes. J. Immunol..

[51] Sanford J.A., Zhang L.J., Williams M.R., Gangoiti J.A., Huang C.M., Gallo R.L. (2016). Inhibition of HDAC8 and HDAC9 by microbial short-chain fatty acids breaks immune tolerance of the epidermis to TLR ligands. Sci. Immunol..

[52] Schwarz A., Bruhs A., Schwarz T. (2017). The short-chain fatty acid sodium butyrate functions as a regulator of the skin immune system. J. Investig. Dermatol..

[53] Williams M.R., Gallo R.L. (2015). The role of the skin microbiome in atopic dermatitis. Curr. Allergy Asthma Rep..

[54] Miller L.S., Cho J.S. (2011). Immunity against *Staphylococcus aureus* cutaneous infections. Nat. Rev. Immunol..

[55] Dreno B., Martin R., Moyal D., Henley J.B., Khammari A., Seité S. (2017). Skin microbiome and acne vulgaris: Staphylococcus, a new actor in acne. Exp. Dermatol..

[56] Chng K.R., Tay A.S.L., Li C., Ng A.H.Q., Wang J., Suri B.K., Matta S.A., McGovern N., Janela B., Wong X.F.C.C. (2016). Whole metagenome profiling reveals skin microbiome-dependent susceptibility to atopic dermatitis flare. Nat. Microbiol..

[57] Simpson E.L., Chalmers J.R., Hanifin J.M., Thomas K.S., Cork M.J., McLean W.I., Brown S.J., Chen Z., Chen Y., Williams H.C. (2014). Emollient enhancement of the skin barrier from birth offers effective atopic dermatitis prevention. J. Allergy Clin. Immunol..

[58] Lodén M. (2003). Role of topical emollients and moisturizers in the treatment of dry skin barrier disorders. Am. J. Clin. Dermatol..

