# Supplementary material for: Pilot Study on the Forehead Skin Microbiome and Short Chain Fatty Acids Depending on the SC Functional Index in Korean Cohorts
Source: Microorganisms. 2021 Oct 25;9(11):2216. doi: 10.3390/microorganisms9112216 (PMC8617931; doi:10.3390/microorganisms9112216)
Supplement: Supplementary file 1 [file microorganisms-09-02216-s001.zip › microorganisms-1415299-supplementary.pdf]

Supplementary Materials

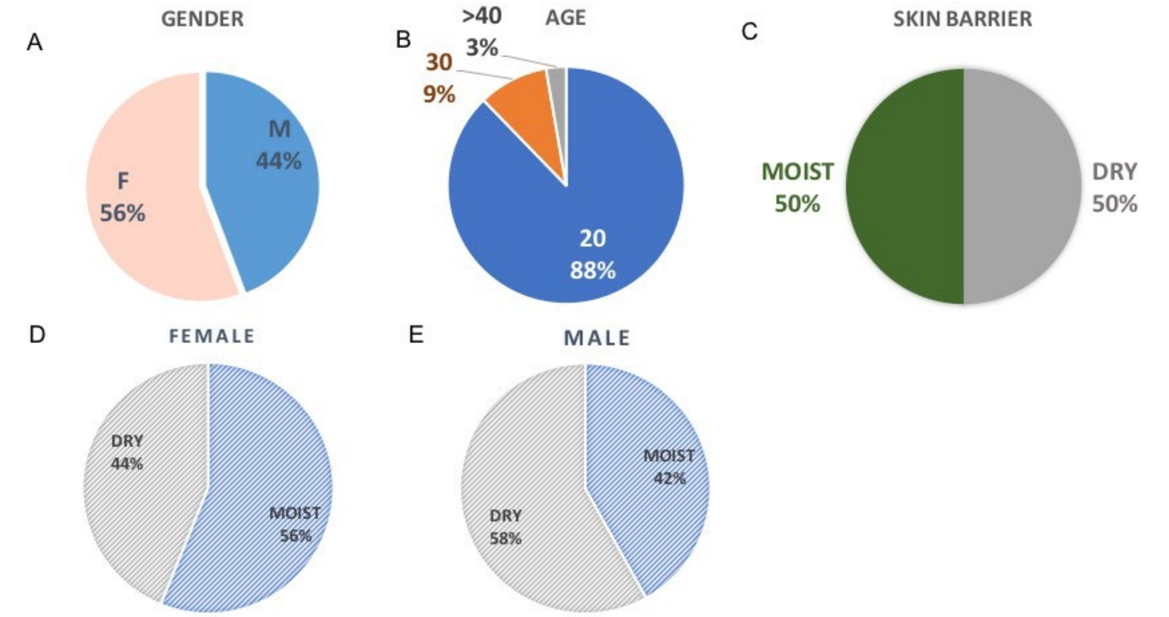

Figure S1. Information on the 150 volunteers for the skin studies. (A) Gender. (B) Age. (C) Skin barrier type. (D) Female skin barrier ratio. (E) Male skin barrier ratio.

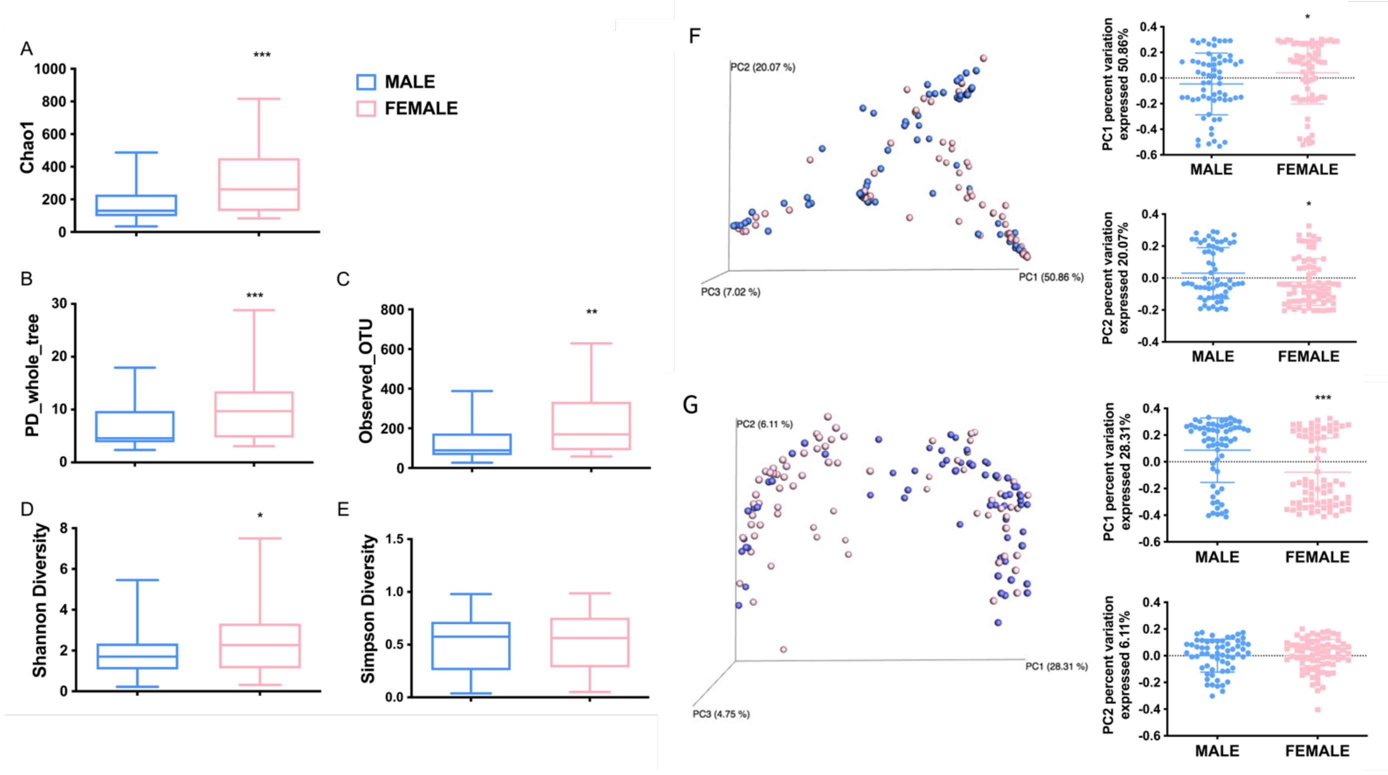

Figure S2. Comparison of gender influence on the diversity of the forehead skin microbiota. (A) Chao 1. (B) PD_whole_tree. (C) Observed_OTUs. (D) Shannon Diversity. (E) Simpson Diversity. (F) Weighted PCoA plot and PC1 and PC2 percent variation. (G) Unweighted PCoA plot and PC1 and PC2 percent variation. (A)-(E) Alpha diversity. (F)-(G) Beta-diversity. Data were analyzed with unpaired parametric t-test with Welch correction compared between genders. *P<0.05, **P<0.01, ***P<0.001.

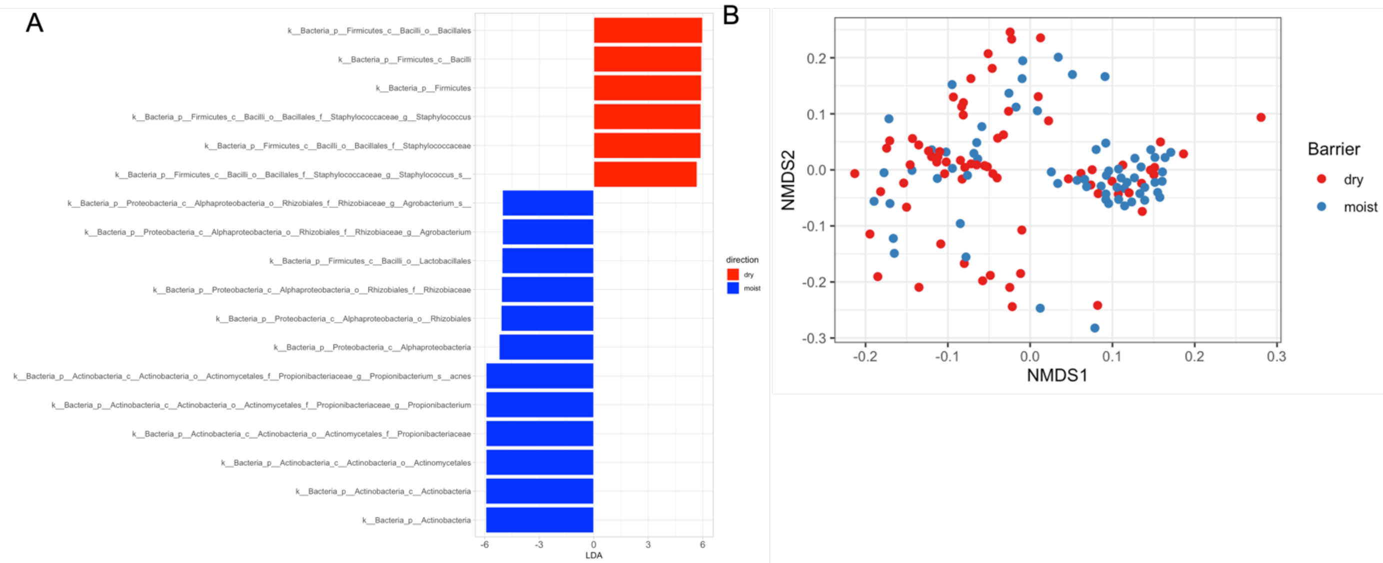

Figure S3. Effect of size and differences in microbial communities on dry and moist skins. (A) Linear discriminant analysis (LDA) and effect Size (LefSe) analysis of microbial communities on dry and moist forehead skins. Positive LDA scores (red bars) are enriched in the dry group and negative LDA scores (blue bars) are enriched in the moist group. The microbial taxa and functions shown in the Figure have a LDA score higher than 5. (B) Bray-Curtis based NMDS results for microbial community groups of dry and moist skins (PERMANOVA, df:1, SS: 0.06199, MS:0.061990, F:5.3909, p-value<0.019).

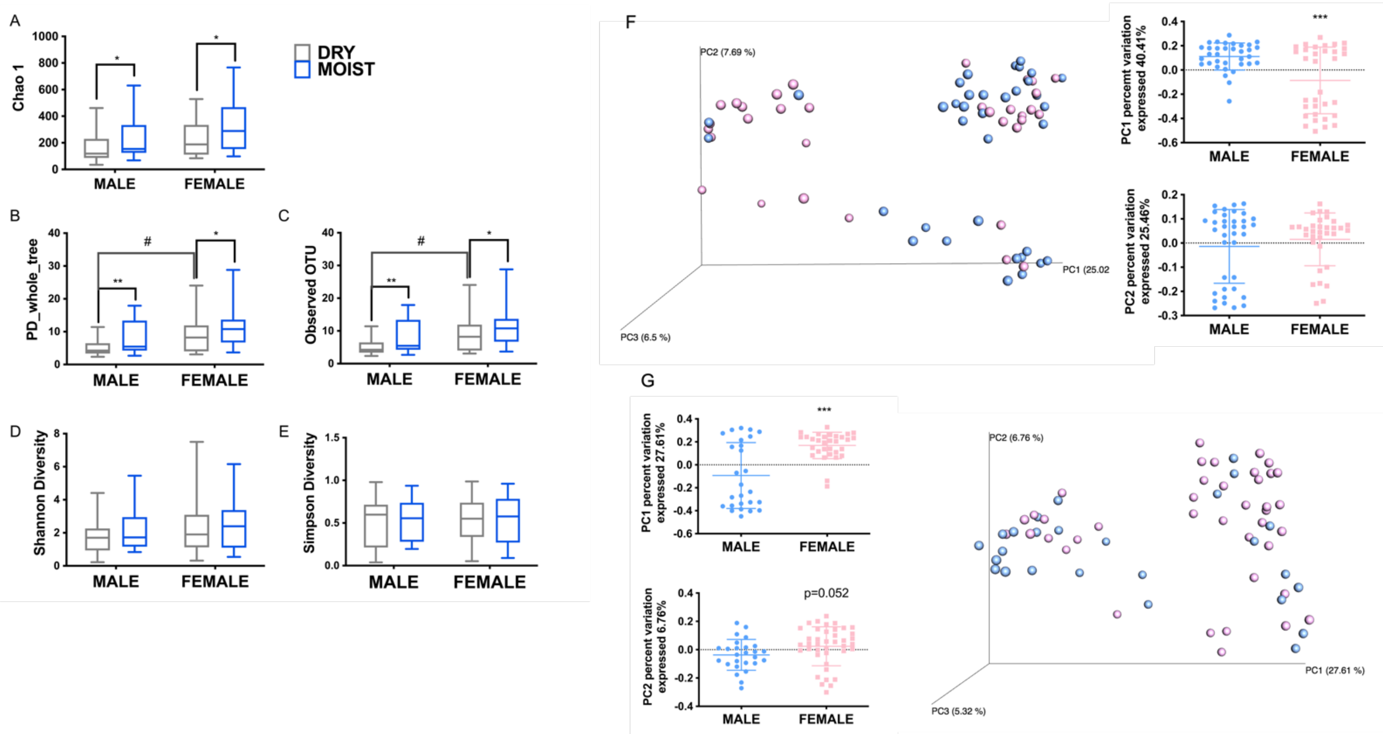

Figure S4. Comparison of gender influence on the diversity of the forehead skin microbiota of dry and moist skin. (A) Chao 1. (B) PD_whole_tree. (C) Observed_OTUs. (D) Shannon Diversity. (E) Simpson Diversity. (F) Dry group unweighted PCoA plot and PC1 and PC2 percent variation. (G) Moist group unweighted PCoA plot and PC1 and PC2 percent variation. (A)-(E) alpha diversity. (F)-(G) beta-diversity. Data were analyzed with unpaired parametric t-test with Welch correction compared with dry group *P<0.05, **P<0.01, ***P<0.001 and between gender #P<0.05.

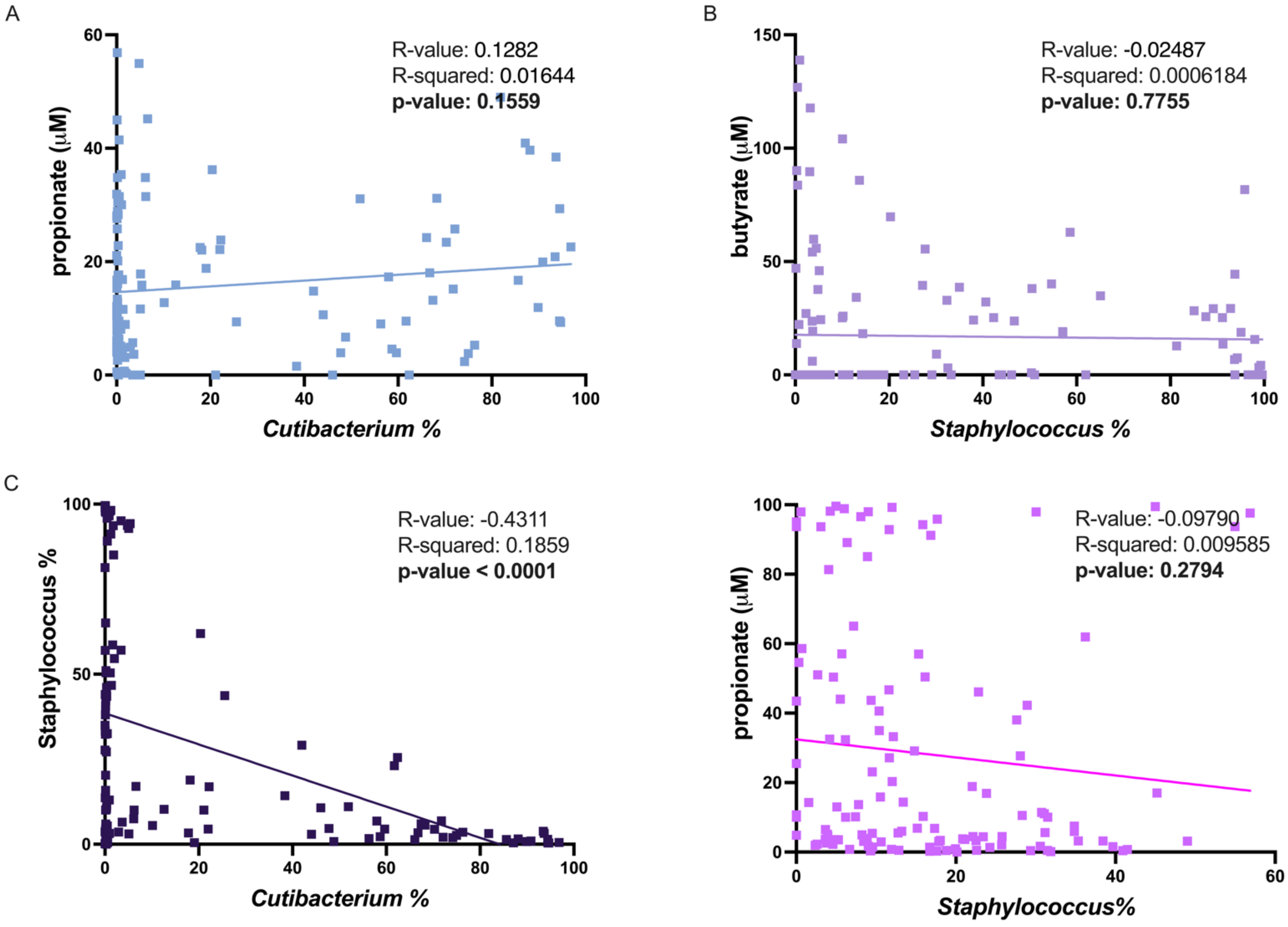

Figure S5. Correlation between the two major bacterial genera and short chain fatty acids (SCFA) of the forehead skin of a cohort of 150 Korean participants. (A) Propionate vs. *Cutibacterium.* (B) Butyrate vs. *Staphylococcus.* (C) *Staphylococcus* vs. *Cutibacterium.* (D) Propionate *vs. Staphylococcus.* Data were analyzed by calculating the Pearson's correlation coefficient.
